# Book Review

**DOI:** 10.1120/jacmp.v7i3.2298

**Published:** 2006-08-24

**Authors:** René J. Smith

**Affiliations:** ^1^ Department of Radiation Oncology The Reading Hospital and Medical Center 6^th^ Avenue & Spruce Street PO Box 16052 Reading PA 19611

## Abstract

Editors Note: This book is written in Spanish and the review is given in both English and Spanish. Both reviews were written by the same individual and essentially have the same content.

*Code of Practice for Quality Control of Treatment Planning Systems (TPS) in Radiation Therapy*, by José Miguel Delgado Rodríguez, Feliciano García Vicente, Esther Millán Cebrián. Sociedad Española de Física Médica. Ramírez de Arellano Editores, S. L. 2005, Madrid, Spain; available. secretaria@sefm.es, 50ε

*Protocolo para Control de Calidad en Sistemas de Planificación de Terapia con Radiaciones Ionizantes*, por José Miguel Delgado Rodríguez, Feliciano García Vicente, Esther Millán Cebrián. Sociedad Española de Física Médica. Ramírez de Arellano Editores, S. L. 2005, Madrid, Spain.

secretaria@sefm.es, 50ε

This volume, written in Spanish, is a welcome addition to Spanish speaking Medical Physicists. I am not aware of a similar book in Spanish that addresses quality control of treatment planning systems. The purpose of this volume is to present in a simple, but complete way the steps to be followed by a clinical radiation oncology physicist during the acceptance and testing of a new treatment planning system. It also recommends quality control procedures to be followed by the user. This volume is not intended to be a final document, but it may be modified and adapted according to different circumstances.

The book is divided into six chapters.

Chapter 1 addresses the information system. It explains the steps necessary to test the new equipment, to verify its functionality and the adequacy of the instruction manuals.

The main objective of chapter 2 is the specification of radiation beams, their position in relation to the patient, the geometric configuration of the unit (teletherapy, as well as brachytherapy units, are considered) and beam modeling.

Chapter 3 explains the steps to be taken to verify the correct acquisition and archiving of images to be used during treatment planning. CT images and digitally reconstructed radiographs (DRR) are discussed.

Beam dosimetry is presented in chapter 4. This chapter deals with the verification of the information needed by the medical physicist to implement correctly the algorithms used by the treatment planning system.

Brachytherapy is addressed in chapter 5. This is not an easy subject, because of the different ways in which the activity of a radioactive source can be specified (kerma, activity contained, apparent activity, mg Ra equivalent). AAPM TG43 is used as the basis of this chapter. This document is almost universally accepted and easily obtainable. Some of the pitfalls of brachytherapy are identified.

Chapter 6, the last chapter, concerns the quality control of digitally reconstructed radiographs. They may come from simulators, or from CT scans. Regardless of their sources, the same care must be taken to avoid image distortion and assure the proper scaling of these images.

The book includes five appendices that complement and expand, in a practical way, some of the material covered in the main text. Each chapter and each appendix contains a fairly up‐to‐date bibliography that includes the most important references used by the authors in the development of the protocol. The volume also includes a two‐page glossary with some of the key words used in the text. The inclusion of an index would have been welcome.

This is a well‐written book that should be very helpful to any Medical Physicist who will go through the testing and acceptance of a new treatment planning system. The authors are to be commended for breaking down very important procedures into a set of understandable instructions.

El protocolo tiene el propósito de presentar en una forma simple, pero completa, los conocimientos requeridos y pasos a seguir por un(a) Radiofísico(a) Hospitalario(a) para aceptar un sistema de planificación antes de ponerlo en uso clínico. Los autores no pretenden que este protocolo sea un documento final, sino que sirva como una guía adaptable a distintas circunstancias para evaluar y aceptar un nuevo sistema de planificación.

El protocolo está dividio en seis capítulos:

1. Sistema Informático. El físico debe conocer el hardware y software que se va a utilizar en el sistema, así como las limitaciones del mismo.
2. Diseño y Modulado de Unidades y Haces de Radiación. En este capítulo se trata la importante tarea de la definición de las características geométricas de la unidad, así como el modelado de los haces de radiaciones ionizantes.
3. Adquisición de Datos Anatómicos. La adquisición y manipulación correcta de imágenes es esencial en un sistema de planificación. Las imágines pueden provenir de distintas unidades, como TC, RM, SPECT, PET.
4. Dosimetría de Haces. La aplicación e interpretación correcta de los algoritmos usados en el sistema de planificación es indispensable para asegurar que el paciente reciba la dosis prescrita.
5. Braquiterapia. Es un área de suma importancia, debido las diferentes unidades en las que la actividad de una fuente radioactiva se puede expresar. Esto puede ocasionar errores en la dosis absorbida por el paciente Los autores siguen las recomendaciones del Grupo de Trabajo # 43 de la AAPM (TG43).
6. Presentación y Transferencia de Datos y Resultados. Es de suma importancia la fidelidad y calidad de las imágenes digitales reconstruídas (RDR), en la verificación y ejecución de un plan en una unidad de teleterapia y braquiterapia.

Cada capítulo está organizado en una forma lógica que explica los pasos a seguir, las pruebas que se deben realizar, el material necesario para llevar a cabo los procedimientos indicados y el análisis de los resultados obtenidos, los valores aceptables y la frecuencia con la cual cada prueba debe repetirse. Al final de cada capítulo hay detalles y advertencias que se deben de tener en cuenta para evitar errores. El documento tiene cinco anexos que complimentan el protocolo.

Cada capítulo y de cada anexo tiene una blibliografía que incluye las referencais más importantes usadas por los autores en el desarrollo del protocolo. La mayoría de las referencias son bastante recientes y fáciles de obtener. El libro contiene un glosario de términos importantes que se usan en el texto del protocolo. El libro no tiene un índice que pudiera facilitar su uso.

Las pruebas a realizar, así como el análisis de los resultados son explícitos y relativamente fáciles de implementar. Los autores han logrado simplificar y exponer de una manera práctica lo que es en realidad una labor de suma importancia desde el punto de vista clínico.

